# Genotype-Dependent Response of Root Microbiota and Leaf Metabolism in Olive Seedlings Subjected to Drought Stress

**DOI:** 10.3390/plants13060857

**Published:** 2024-03-15

**Authors:** Rahma Azri, Myriam Lamine, Asma Bensalem-Fnayou, Zohra Hamdi, Ahmed Mliki, Juan Manuel Ruiz-Lozano, Ricardo Aroca

**Affiliations:** 1 Laboratory of Plant Molecular Physiology, Centre of Biotechnology of Borj-Cedria, P.O. Box 901, Hammam-Lif 2050, Tunisia; 2 National Insitute of Applied Science and Technology, University of Carthage, Centre Urbain Nord, BP 676, Charguia Cedex 1080, Tunisia; 3 Departament of Microbiology, Soil System and Symbiosis, Zaidín Experimental Station, Spanish Reaserch Council (CSIC), Prof. Albareda 1, 18008 Granada, Spain

**Keywords:** olive genotype, metabolomics, indigenous and exogenous microbes, rhizosphere, bulk soil, drought stress

## Abstract

Under stress or in optimum conditions, plants foster a specific guild of symbiotic microbes to strengthen pivotal functions including metabolic regulation. Despite that the role of the plant genotype in microbial selection is well documented, the potential of this genotype-specific microbial assembly in maintaining the host homeostasis remains insufficiently investigated. In this study, we aimed to assess the specificity of the foliar metabolic response of contrasting olive genotypes to microbial inoculation with wet-adapted consortia of plant-growth-promoting rhizobacteria (PGPR), to see if previously inoculated plants with indigenous or exogenous microbes would display any change in their leaf metabolome once being subjected to drought stress. Two Tunisian elite varieties, Chetoui (drought-sensitive) and Chemleli (drought-tolerant), were tested under controlled and stressed conditions. Leaf samples were analyzed by gas chromatography–mass spectrometry (GC-TOFMS) to identify untargeted metabolites. Root and soil samples were used to extract microbial genomic DNA destined for bacterial community profiling using 16S rRNA amplicon sequencing. Respectively, the score plot analysis, cluster analysis, heat map, Venn diagrams, and Krona charts were applied to metabolic and microbial data. Results demonstrated dynamic changes in the leaf metabolome of the Chetoui variety in both stress and inoculation conditions. Under the optimum state, the PGPR consortia induced noteworthy alterations in metabolic patterns of the sensitive variety, aligning with the phytochemistry observed in drought-tolerant cultivars. These variations involved fatty acids, tocopherols, phenols, methoxyphenols, stilbenoids, triterpenes, and sugars. On the other hand, the Chemleli variety displaying comparable metabolic profiles appeared unaffected by stress and inoculation probably owing to its tolerance capacity. The distribution of microbial species among treatments was distinctly uneven. The tested seedlings followed variety-specific strategies in selecting beneficial soil bacteria to alleviate stress. A highly abundant species of the wet-adapted inoculum was detected only under optimum conditions for both cultivars, which makes the moisture history of the plant genotype a selective driver shaping microbial community and thereby a useful tool to predict microbial activity in large ecosystems.

## 1. Introduction

In recent decades, the area of irrigated olive groves has increased dramatically in the Mediterranean area. The intensification of cultures in the drip watering system mostly replaced the rain-fed mode (63%) [1]. The effects of climate change, particularly drought and heat waves, become more pronounced in affecting the oleiculture sector [2]. However, the olive species are well adapted to water deficiency [3]; intense and extended periods of drought led to significant decline in their growth and productivity [4]. To address the loss, a multitude of crop resistance amelioration projects were launched [5]. Breeding programs opt to compare the abiotic stress response of wild and domesticated genotypes to select for resilient cultivars [6]. The results of related experiments demonstrated that plants’ intrinsic capacity to mitigate stress is mainly supported by microbial endophytes, which play a crucial role in maintaining their homeostasis [7]. In natural ecosystems, plants foster a specific guild of soil microbes to accomplish pivotal functions [8]. Their system deploys specific signaling molecules to discriminate beneficial microbes from the pathogenic strains to be repelled [9]. Establishment of such interaction with favored microbial clusters is influenced by a myriad of factors involving the environmental conditions experienced by both partners [10]. Strong evidence explained how the autochthonous microbiome and the native plant species belonging to the same biotope have undergone a long co-evolution process resulting in strong genomic interdependency [11]. A high-throughput analysis emphasized that soil microbes are transmitted to the following plant generation from which they acquire a new subset of functional genes under the mechanism of horizontal gene transfer [12]. This tight linkage among the holobiont indicates that plant microbe recruitment is not random; it is rather controlled by a strict set of rules [13]. Robust findings revealed that the transcriptional profile of selected microbes undergoes the utmost shaping by plant genetics [14]. To tackle stress, a plant produces an array of metabolites regulating its biochemistry and physiology [15]. The origin and composition of endosphere microbes, considered as an extension of the plant phenotype, has a strong influence on the plant metabolome [16]. Compared to roots, the foliar chemistry can be more effective to represent stress-driven perturbation on plant status because leaves are directly exposed to dry and heat waves. However, plant leaves and roots follow distinct developmental trajectories; they coordinate mechanisms in response to drought and microbial signaling induction. The coordination of metabolic assimilation is a key factor for plants’ adaptive mechanisms to drought stress. For plants, water availability in soil is a major limiting factor for growth and productivity. To acclimate to drought conditions, leaves develop fine-tuning energy production when water and nutrients are unavailable [17]. Root inoculation with symbiotic microbes including plant-growth-promoting bacteria (PGPR) can directly affect leaf metabolic integrity [18]. Their impact is conditioned by the degree of interaction with the host plant [19]. Microbial consortia stimulate the production of biomolecules that induce systemic tolerance to drought [20]. This mechanism involves cellular, molecular, and biochemical modifications [21]. The microbial consortia obtained from extreme environments are endowed with a great potential to support the systemic tolerance of drought-sensitive varieties, though they are adapted to low resources [22]. However, they might change crop characteristics and engender the loss of biodiversity in native communities [23]. Accordingly, novel studies start focusing on engineering the holobiont as a hole instead of applying exogenous microbes from different environments [24]. In General, the approach harnessing the tolerant microbiome to support the sensitive varieties subjected to drought stress suffers serious flaws proven by open field failure of exogenous microbiome application: plant immune barriers will consider the introduced microbes as foreigner organisms that should be chemically repelled by root exudates. In addition, the microbial activity of newcomers can be suppressed by the already established resident community. Incompatibility between host plants and the introduced microbes (chemically and genetically) may cause a dysfunction for both. Exogenous microbe application might also change and degrade plant organoleptic characteristics specifically in terroir products like olive and wine. For so long, the microbiome of sensitive varieties has been considered unqualified to support plants in mitigating stress. Recently, new experiments have proven that training the wet-adapted microbes by recurrent drought can enhance their adaptability traits and render them tolerant. In fact, manipulating and engineering the microbial local communities will be a more accurate and cost-effective agronomic approach.

The current work was aimed to compare the microbial-mediated change in the leaf metabolome of drought-sensitive (conspecific) and drought-tolerant (heterospecific) olive genotypes under stress and in an optimum condition. Seedlings of our experiment were grown in an agronomic soil obtained from an olive grove to mimic open field conditions. The inoculation occurred using the autochthonous microbial consortium of the sensitive variety to capture a co-evolving set of PGPRs that colonize olive in nature. To test whether the plant genotype can engender a fingerprint microbial–metabolic response to drought stress, we ran untargeted metabolomic profiling using the gas chromatography–mass spectrometry (GC-TOFMS) technique and a microbial analysis using 16S metagenomics. Differential and shared bacterial–metabolic features within contrasting genotypes would give insights into drought response improvement for sensitive crops.

## 2. Results

In order to investigate the influence of microbial inoculation with wet-adapted PGPRs on the primary metabolism of their corresponding host (the olive-sensitive cultivar) and a genetically distant host (the olive-tolerant cultivar) maintained under water stress during three years experiment, we analyzed both the root entophytic bacteria and the leaf metabolic profiles in triplicate for four treatments.

### 2.1. Assignment of Significant Metabolic Biomarkers Related to Stress and Inoculation

**Figure 1:**
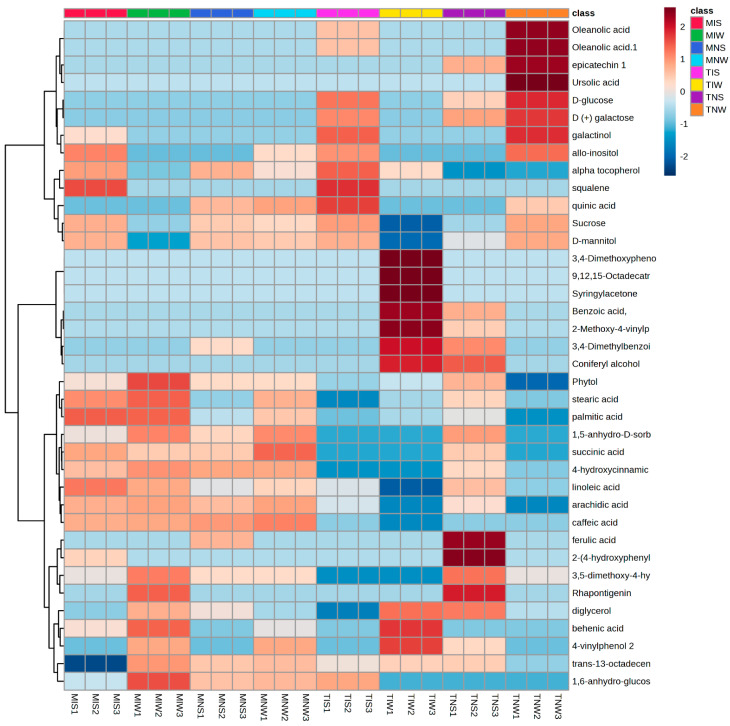
Heat map illustrating the most significant leaf metabolites of two olive genotypes subjected to drought stress.

Using the large multidimensional data sets generated with GC-MSTOF and an amplicon sequencing analysis, we constructed heat maps that illustrate differences in the relative concentration of foliar metabolites in olive tissues subjected to drought and PGPR inoculation. We identified metabolic features of both cultivars maintained under similar and different experimental conditions (Figure 1). The great part of the identified molecules covered the primary metabolism pathways. Classification comprises seven major groups: fatty acids, tocopherols, phenols and methoxyphenols, stilbenoids, triterpens, and sugars. The rows of metabolic compounds were hierarchically clustered to allow the grouping of similar patterns into specific sample associations with annotated metabolites. Data are represented in a grid with rows and columns for metabolic compounds and their corresponding treatments. The color key in the top right of the heat map shows the illustration of the variation of metabolic abundance ranging from a blue (−) to red shade (+). The distribution of overall metabolic compounds in different cultivars was uneven. The Chemleli variety displayed comparable metabolic profiles among treatments. Fatty acid compounds (stearic acid, palmitic acid, linoleic acid, and arachnid acid) were highly accumulated in MIS, MIW, and MNW except in stressed, non-inoculated plants (MNS). A significant increase in 3,5-dimethoxy-4-hydroxycinnamic acid, rhapontigenin, diglycerol, behenic acid, 4-vinylphenol 2, trans-13-octadecenoic acid, and 1,6-anhydro-glucose was remarked in the optimum condition (MIW). Oleanolic acid, epicatechin, ursolic acid, D-glucose, and D (+) galactose were appearing in low abundance. Similar patterns were detected for the majority of phenols and methoxyphenols (3,4-Dimethoxyphenol, 9,12,15-Octadecatrienoic acid, syringylacetone, benzoic acid, 2-Methoxy-4-vinylphenol, and 3,4-Dimethylbenzoic acid). Under stress, the PGPR inoculation decreased the concentration of 3,5-dimethoxy-4-hydroxycinnamic acid, rhapontigenin, diglycerol, behenic acid, 4-vinylphenol 2, trans-13-octadecenoic acid, and 1,6-anhydro-glucose yet enhanced the accumulation of more triterpenes (alpha tocopherol, squalene) and sugars (galactinol, allo-inositol, sucrose, and D-mannitol) in both genotypes. The Chetoui cultivar exhibited distinctive metabolic partitioning. Without the inoculum, stressed plants tend to strongly accumulate some phenols and methoxyphenols (ferulic acid, 2-(4-hydroxyphenyl) ethanol, 3,5-dimethoxy-4-hydroxycinnamic acid) but slightly accumulate others (benzoic acid, 2-Methoxy-4-vinylphenol and 3,4-Dimethylbenzoic acid, coniferyl alcohol, phytol, 4-hydroxycinnamic acid, and succinic acid). In the optimum condition (TIW), the phenolic compounds (3,4-Dimethyphenol, 9,12,15-Octadecatrienoic acid, syringylacetone, benzoic acid, 2-Methoxy-4-vinylphenol, and 3,4-Dimethylbenzoic acid) were present in significant concentrations unlike in TIS and TNS and the control treatment (TNW) where metabolites were almost depleted. An important increase was only detected in sugars and fatty acids (oleanolic acid, epicatechin, ursolic acid, D-glucose, D (+) galactose, galactinol, allo-inositol, sucrose, and D-mannitol). The Chemleli cultivar preserved relatively stable patterns. In different treatments, there was not much variation in polyphenols, sugars, or fatty acids. The genotype was not affected by stress nor by PGPR inoculation. That distinguished behavior might be attributed to its intrinsic robustness. In contrast, the Chetoui variety demonstrated clear disparities between metabolic profiles of inoculated and non-inoculated treatments under well-watered and stress conditions (highly abundant fatty acids and sugars remarked in TNW treatment and highly abundant polyphenols recorded for TIW). In stressed leaves, the metabolic profiles seemed to be inversed. Such a specific response where the metabolome variation is solely being explained by the impact of microbial inoculation on their biochemistry demonstrates high sensitivity of the Chetoui genotype to the application of indigenous consortia.

### 2.2. Matching Olive-Genotype-Dependent Response to Drought and PGPR Application

**Figure 2:**
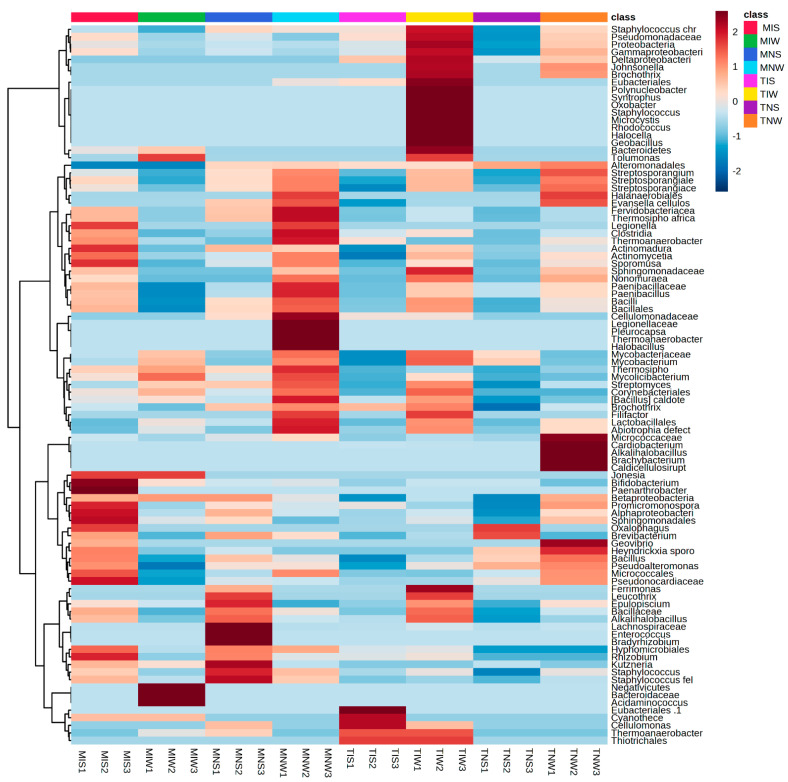
Heat map with relative abundance of bacterial taxonomic groups colonizing two olive genotypes under drought and control conditions.

To identify the bacterial community colonizing the root system, we followed the same steps as metabolic characterization (Figure 2). For different treatments of the *Terrabacteria* clade, the *Actinomecetia* and *Firmicutes* were the most dominant phyla. Under the hierarchical cluster, we classified the PGPRs at family and genus levels. In the four treatments of the Chemleli variety, the phylum of *Proteobacteria* (*Syntrophus* and *Tolamonas* classes), Cyanobacteria (*Mycrocystis*), *Oxobacter*, and *Johnsonella* genera was present in low abundance. The roots of stressed seedlings tend to recruit *Thermosiphoafricanus*, *Legionella*, *Clostridia*, *Thermoanaerobacter*, *Actinomadura*, *Sporomusa* and *Bacillus*. In MIS treatment, we remarked a significant proliferation of *Jonesia*, *Bifidobacteruim*, *Paenarthobacter*, *Betaproteobacteria, Promicromonospora*, *Alphaproteobacteria*, *Sphingomonadales*, *Oxalophagus*, *Brevibacteruim*, *Bcillus*, *and Micrococcales. Rhizobuim*, *Kutzneria*, and *Staphylococcus* were also highly abundant. The drought-tolerant cultivar appears to be indifferent to the addition of a wet-adapted inoculum where almost all of the PGPRs appeared in the trace except *Bacteroidaceae*, *Acidaminococcus*, *Mycobacteriacea*, and *Streptomyces*. Surprisingly, non-inoculated plants (MNS and MNW) tend to accumulate more bacteria from the bulk soil serving as a planting substrate instead of the rhizospheric inoculum of the Chetoui variety, which proposes that incompatibility of contrasting lifestyles between the inoculum and the host plant can restrain the colonization process. The profiles of bacteria colonizing the root of non-inoculated seedlings differ significantly between stressed and non-stressed treatments. In MNS, the most abundant taxa were *Epilopiscium*, *Bacillaceae*, *Alkalihalobacillus*, *Lachnospiraceae*, *Enterococcus, Bradyrhizobuim*, *Hyphomicrobiales*, *Rhizobuim*, *Kuznzeria*, and Staphylococcus. The unique microbial fingerprint was recorded in optimum status with great abundance of *Fervidobacteriaceae*, *Thermosiphoafricanus*, *Legionella*, *Clostridia*, *Paenobacillus*, *Cellulomondaceae*, *Pleurocapsa*, *Hallobacillus*, *Fillifactor*, *Lactobacillus*, and *Abiotrophia defect*, suggesting that moisture history of the inoculum may play a crucial role in microbial performance. For the Chetoui cultivar, the well-watered condition seemed to ramp up bacterial proliferation, and the inoculation with the indigenous consortium of rhizospheric soil increased the abundance of *Staphylococcus*, *Pseudomonadaceae*, *Gammaproteobacteria*, *Deltaproteobacteria*, *Johnsonella*, *Brochothrix*, *Eubacteriales*, *Polynucleobacter*, *Syntrophus*, *Oxobacter*, *Staphylococcus*, *Mycrocystis*, *Rhodococcus*, *Halocella*, *Geobacillus*, *Bacteroidetes*, and *Tolumonas* to a maximum level. High bacterial colonization was also detected for *Sphingomonadaceae*, *Mycobacteriaceae*, *Brochotryx*, *Filifactor*, *Ferrimonas*, *Leucothrix*, *Epulopiscium*, *Thermoanaerobacter*, and *Thiotrichales*. The non-inoculated, non-stressed seedlings (TNW) acquired a specific subset of bacterial species from the bulk soil (*Micrococcaceae*, *Cardiobacterium*, *Alkalihalobacillus*, *Brachybacteruim*, *Caldicellulosirupt*, and *Geovibrio*) and some common bacterial features with TIW like *Staphylococcus*, *Pseudomondaceae*, *Protebacteria*, *Gammaproteobacteria*, *Deltaproteobacteria*, *Johnsonella*, *Brochothrix*, *Aleromondales*, *Streptosporanguim*, *Achtinomycetia*, *Sphingomondaca*, and *Nonomurae.* These common taxa might be a shared core microbiome between bulk and rhizospheric soil of the same plant species. Under stress, a low level of colonization was recorded in TIS and TNS; it appears that microbial recruitment burdens the drought-sensitive variety that holds a position of supplying photosynthetic products in exchange for water and nutrients. The fact that the great bacterial abundance was only remarked in well-watered treatments proves the importance of soil moisture legacy in host–microbe interaction.

### 2.3. Metabolic Profiling of PGPR-Induced Changes in Olive Tissues

**Figure 3:**
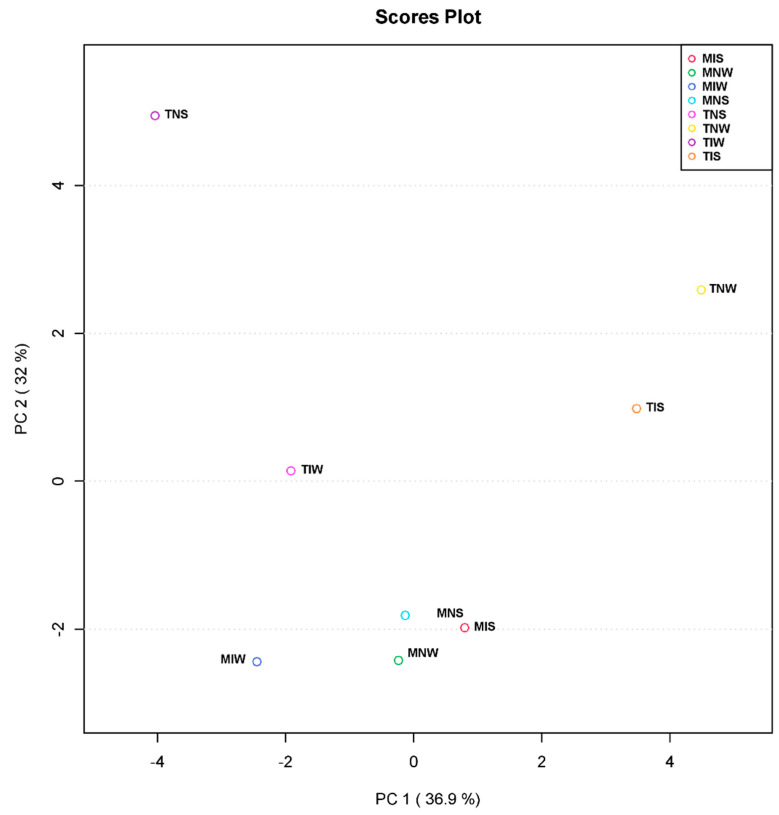
Principal component analysis (PCA) based on leaf metabolite profiles of Chetoui and Chemleli olive cultivars subjected to drought stress.

For further understanding of the microbial-induced change in two genotypes subjected to drought stress at the metabolic level, we performed a principal component analysis. PCA served to reduce data dimensionality and facilitate the visualization of the relationship linking the treatments (Figure 3). The first axis (PC1) explained 36.9% of total variation, and the principal component PC2 explained 32% of variation across the data set. The score plot of PC1 and PC2 revealed a clear separation between samples of the sensitive variety. The non-inoculated, stressed treatment (TNS) occupied a distinctive position of the score plot. Being the most distant from TIS and TNW means that their leaf metabolic differences were the greatest. In contrast, samples of the tolerant variety and TIW treatment represented a small variation along horizontal and vertical axes (Y, X). The distance between MNS, MIS, and MNW and the well-watered treatment of the tolerant variety (MIW) showed that its metabolome was highly altered by PGPR inoculation. We can retrieve from these results that the wet-adapted inoculum is potentially functional under optimum conditions and that the metabolism of sensitive cultivars can be assimilated to the tolerant variety if they are inoculated with their conspecific consortium.

**Figure 4:**
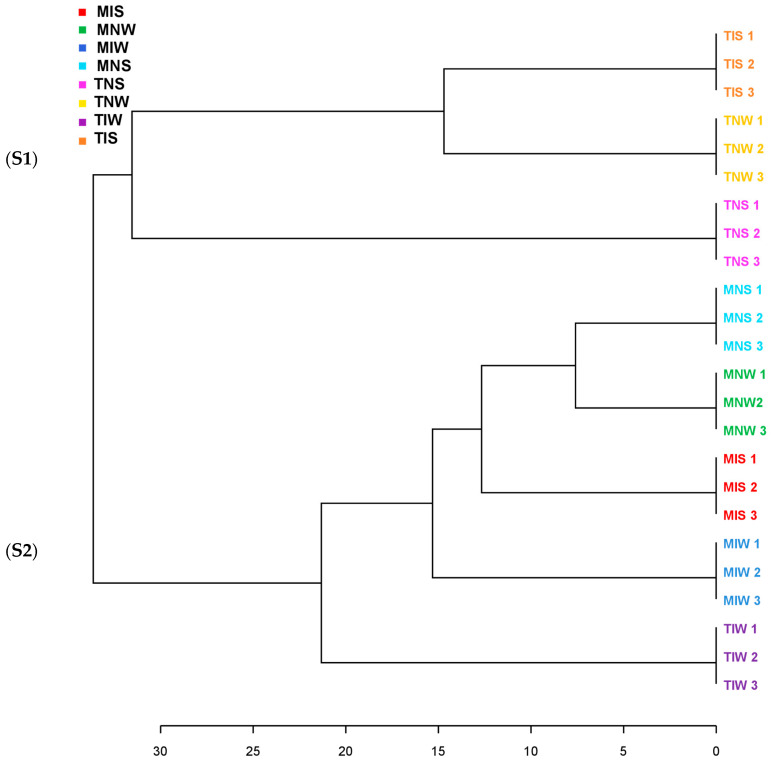
Clustering pattern displaying the dendrogram of two olive genotypes with different drought sensitivity maintained under water stress and microbial inoculation (represented leaf metabolites were segregated in two subclusters S1 and S2).

**Figure 5:**
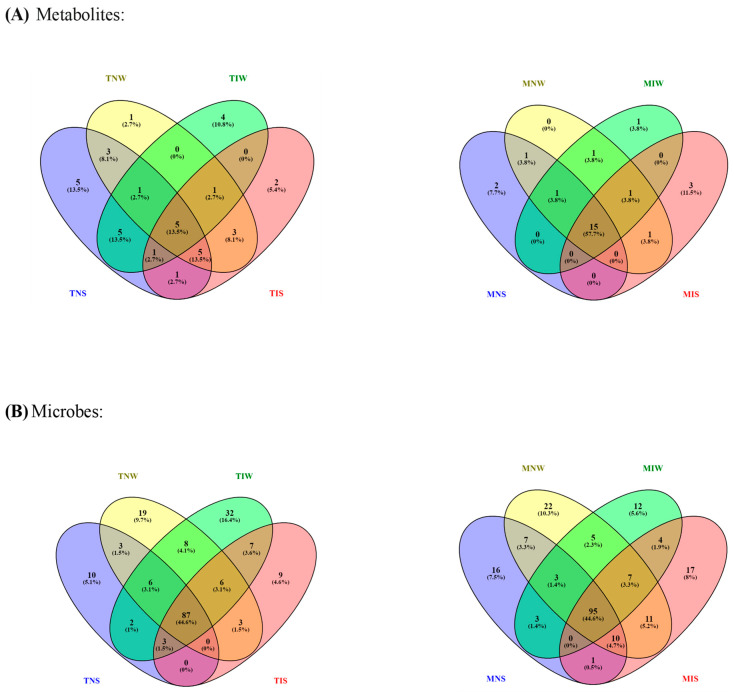
Venn diagrams of shared–unique metabolic (**A**) and microbial features (**B**) in olive cultivars subjected to drought stress. Different color belongs simply to the treatments.

We ran a dendrogram analysis to confirm previous findings (Figure 4). Leaf metabolites of both genotypes were scattered in two main clusters. First, subcluster S1 comprises the TIS, TNW, and TNS samples of the Chetoui variety. S1 was split into under-subclusters gathering the control (TNW) and the stressed treatment (TIS). This subdivision demonstrated that under stress, the indigenous inoculum of the sensitive cultivar can bring the leaf metabolome near the state of well-watered conditions. The Chemleli samples and the TIW treatment of the Chetoui variety blend under a second subcluster (S2). Similarities in leaf metabolic profiles were detected in stressed (MNS) and non-stressed plants (MNW) when seedlings are not inoculated, which proves that drought has an insignificant effect on leaf biochemistry of tolerant cultivars. In optimum status, TIW and MIW treatments sharing the same subcluster were endowed with comparable metabolic patterns. In fact, the wet-adapted microbiome associated with their specific host was able to change the sensitive variety metabolome to match the foliar chemistry of the drought-tolerant cultivar. Venn diagrams were plotted to highlight shared and unique features in various treatments (Figure 5). As previously stated, the Chemleli variety displayed a neutral response to exogenous factors. More than 50% of plant metabolites (lipids, sugars, phenols, and methoxyphenols) were common between the treatments. Less than 10% of the identified molecules were sample-oriented. Unlikely, the TNW, TNS, TIS, and TIW treatments of the Chetoui cultivar shared only 13% of metabolic entities including lipids, trans-13-octadecenoic acid, and alpha tocopherol. Other compounds were specifically distributed. Hence, the condition (inoculated/non-inoculated, stressed/non-stressed) of sensitive plants is deemed to be a determinant in leaf metabolic composition. The bacterial taxa *Fervidobacteriaceae*, *Bacillaceae*, *Paenibacillaceae*, *Micrococcaceae*, *Pseudonocardiaceae*, *Streptosporangiaceae*, *Paenibacillaceae*, *Lachnospiraceae*, and *Pseudomonadaceae* were shared in a proportion of 44% between the treatments in both varieties. The Chetoui endosphere accumulated more specific taxa (16%) in TIW treatment where the MNW roots selected only 10% of specific PGPRs. The common features colonizing both cultivars might be explained by genomic redundancy of certain microbial taxa resident in the rhizosphere and bulk soil of the same environments and the clear differences in microbial segregation might be referred to host–genotype affinity.

### 2.4. Soil Characterization

**Figure 6:**
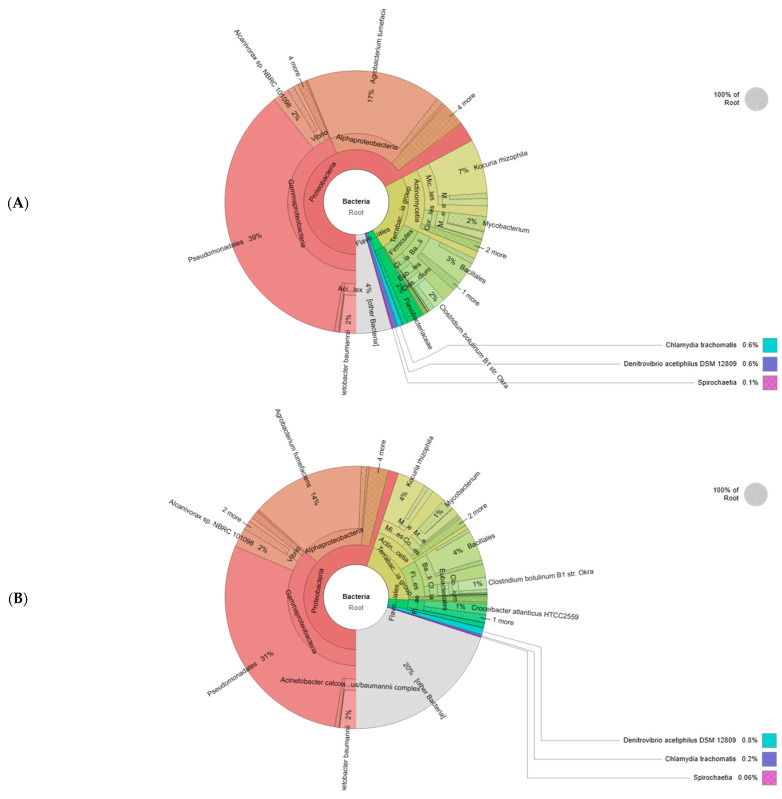
Krona charts with bacterial relative abundance of rhizosphere (**A**) and bulk soils (**B**).

A high-throughput analysis was performed to identify the bacterial community in soil consortia (Figure 6). Over 98% of sequences were mapped into 133,507 reads for the rhizosphere and 105,097 reads for the bulk soil within a 4% and 20% rate of unassigned sequences (microbial dark matter). The Proteobacteria phylum exhibited the highest relative abundance, averaging 67% in the rhizosphere and 55% in bulk soil. Respectively, the *Terrabacteria* group represented 24% and 21% of microbial communities. The *Actinomycetia* class was found in a higher percentage in the rhizosphere (61%) compared to the bulk soil (53%) while the *Firmicute* phylum was dominant in bulk soil (42%) and appeared in lower abundance in the rhizosphere (37%). The *Bacilli* class represented around 70% of both consortia. *Pseudomonadales* was a major order of the rhizosphere consortium (39%) and bulk soil (31%) followed by *Micrococcales* (10% and 6%). The *Flavobacteriales* rate was around 3% in both communities. *Clostridia*, *Corynebacteriales Eubacteriales*, *Enterobacterales*, and *Corynebacteriale Flavobacteriales Burkholderiales* were present in all samples but at a low percentage. At the genus level, *Clostridium*, *Pseudomonas*, *Mycobacterium*, and *Burkholderia* were the most abundant taxa colonizing the rhizosphere. The data presented in Table 1 suggested that the soil characteristics were affected by plant microbe interaction. Compared to the bulk soil, the rhizosphere presented the highest values of nutrients (18.98 g/kg of carbon, 85.21 mg/kg of total nitrogen, and 63.34 mg/kg of available phosphorus). Intense microbial activity and root plant exudates may justify the increased electric conductivity, pH, and humidity augmentation in this zone. However, the low relative abundance of bacterial taxa and the absence of root systems can explain the degraded physicochemical proprieties in bulk soil.

## 3. Materials and Methods

### 3.1. Soil Sampling

Engineering custom effective microbial inoculants for drought-susceptible cultivars imply to understand first the strategy of sensitive varieties in microbial selection under different conditions (stress/optimum) and with different sources (related/distant microbes: rhizosphere and bulk consortia). Thus, to better investigate how the interaction between indigenous microbes, their host, and a genetically distant variety is impairing plant phytochemical composition, we compared the leaf metabolome and microbial profiles of two different olive genotypes subjected to drought stress. Bulk and rhizosphere soil consortia were collected in September 2018 from an olive grove of Chetoui (drought-sensitive variety) located in north Tunisia with geographic coordinates (36°41′ N, 10°18′ E). The sampling site was split into 6 plots to cover the maximum of the pan microbiome. Composite samples of ten cores each containing 100 g of soil were collected at a 5 m distance from the trees (bulk) and 25 cm depth around lateral roots (rhizosphere). Composite samples were mixed, homogenized, sieved (2 mm mesh), and then transferred into sterile iceboxes and transported to the lab to be stored at −20 °C for a further process.

### 3.2. Physicochemical Analysis

We used Mathieu and Piletain’s [25] modified method to assess electric conductivity (EC) in a 1:5 soil/water mix, shaking overnight, filtered and measured with a conductivity meter (WTW inoLab Cond 7110, Germany). Soil pH was determined according to the FIBL handbook using 1:2.5 (*w*/*v*) of a soil/water solution stirred and suspended in 0.01 M CaCl_2_ then measured using a precise pH meter (Sartorius PB10, Germany). Soil moisture (H%) was calculated in 5 g of fresh and oven-drying (105 °C) subsamples following the formula $\frac{fresh\;weight-dry\;weight}{dry}weight\times 100$. Soil organic carbon (C) was determined using K_2_Cr_2_O_7_ volumetric titration following the Walkley–Black external heating method. Total nitrogen (TN) was analyzed by Kjeldahl digestion and the available phosphorus content (Available P) was determined using the sodium bicarbonate extraction and colorimetric assay.

### 3.3. Experimental Design

In this experiment, we used plantlets of Tunisian pioneer olive varieties, Chemleli (drought-tolerant) and Chetoui (drought-sensitive), produced through green budding. Uniform seedlings were selected and each transplanted into a plastic pot (15.0 ± 2 cm diameter) filled with 8 kg of bulk soil. A total of 200 g of clay pellets was added to the bottom of the pots to ensure water draining. Plots were arranged in a randomized complete block design at the greenhouse benches. In total, 200 g of an inoculum of the Chetoui variety was placed in the planting holes around the roots at the time of transplanting. Control pots of non-inoculated plants were prepared using bulk soil as a substrate. For both varieties, treatments were conducted in five replicates. At the early stage (first-year seedlings), the plants were irrigated once per week with 250 mL of distilled water and a diluted (1/4) Vilmorin nutritive solution (20% P, 40% N, and 40% K) to maintain a low N:P ratio in order to promote rapid colonization. After 2 years, we applied moderate stress (non-lethal) at 50% CC and seedlings were receiving a dose of 500 mL of distilled water free of a nutritive solution once per month during 3 months. Non-stressed plants were irrigated at 100% CC twice per week.

The following presents the combination of treatments:

**TNS:** non-inoculated, stressed Chetoui plants

**TNW:** non-inoculated, non-stressed Chetoui plants

**TIW:** inoculated, non-stressed Chetoui plants

**TIS:** inoculated, stressed Chetoui plants

**MNS:** non-inoculated, stressed Chemleli plants

**MNW:** non-inoculated, non-stressed Chemleli plants

**MIW:** inoculated, non-stressed Chemleli plants

**MIS:** inoculated, stressed Chemleli plants

### 3.4. Untargeted Metabolomics

During the harvest, fully expanded olive leaves were collected and grinded in liquid nitrogen. In total, 100 g of the fine powder was weighed and stored in 2 mL tubes in −80 °C for a metabolic analysis. During the extraction, 1 mL of a cold solvent mixture (water/chloroform/methanol ratio, 1:1:2.5 *v*/*v*/*v*) along with 0.2 g/L of an internal standard (α-cholestane, stock concentration: 0.2 mg/mL) was added to the ground leaf material. The mixture was vortexed for 1 min and then kept on ice for 30 min with intermittent shaking. Afterward, the cooled homogenate was centrifuged at 15,000× *g* rpm and 4 °C for 5 min. The collected supernatant was transferred to a new tube, and 200 µL of chloroform and 500 µL of deionized water were added to the mix then vortexed. The tube was then centrifuged at 15,000× *g* rpm and 4 °C for 5 min. This step leads to the separation of the solution into upper (aqueous) and lower (organic) phases. The aqueous phase was dried nitrogen air and subsequently derivatized for GC–MS. The dried aqueous phase was dissolved in 60 µL of a methoxylamine hydrochloride (M.HCL) solution (40 mg/mL in pyridine), and the mix was incubated for 30 min and centrifuged at 15,000× *g* for 5 min then transferred to glass autosampler vials for a subsequent analysis. Prepared samples were injected into a GC analyzer (Agilent 7890B GC unit coupled to a Bench TOF-Select™ system, Llantrisant, UK). For identity confirmation, hard and soft ionizations were set at 70–12 Ev to explore spectral and response complementarity. Ion source temperatures and the front inlet were both kept at 250 °C. The MS optimization option was set to operate in Tandem Ionization™ at a full scan mode with a mass range of *m*/*z* 45–1000 m, 50 Hz acquisition frequency, and filament voltage of 1.50 V. A DB-1 GC column (Agilent Technologies) of a 20 m length, 0.18 mm internal diameter, and 0.18 µm film thickness was mounted with helium carrier gas of a 1 mL/min flow rate. The injector was set to a 1:10 split ratio, with a total injection volume of 1 µL. The oven temperature was programmed in two cycles: 70 °C (2 min) to 120 °C at 10 °C min^−1^, and then to 320 °C (1 min) at 4 °C min^−1^. For the whole samples, 2.0 µL of the derivatized solution was analyzed under the following conditions: a split/spitless injector in split mode, split ratio of 1:20, and injector temperature of 300 °C.

### 3.5. GC-TOF/MS Data Processing

The previously generated chromatograms were treated with ChromaTOF software (version 4.32). The spectra were exported to the program to make baseline corrections, and adjust the retention rate and retention time. Deconvolution, peak identification, and alignment were processed using version 11 of the NIST library. Further metabolic annotation was held using online databases to search for matching compounds. A downstream analysis was performed according to [26]. The ANOVA-generated data were log-transformed, and the significant values were scaled and clustered into a heat map and dendrogram. We performed a principal component analysis (PCA) to separate and classify the sample groups. The Venn diagram was built to identify the shared and different metabolic features among treatments.

### 3.6. Microbial Sequencing and Bioinformatic Analysis

Total genomic DNA was extracted from soil and root samples of both olive genotypes using a DNeasy PowerSoil Kit (QIAGEN, Hamburg, Germany), as described by the manufacturer. The concentration and purity of DNA were examined by Nanodrop (Denovix, USA) and the DNA quality was detected by 1% agarose gel electrophoresis. The PCR amplification was performed using specific barcoded primers for 16S rRNA targeting the V3–V4 region—341 F: CCTAYGGGRBGCASCAG and 806 rB: GGACTACNNGGGTATCTAAT. The library construction and sequencing steps were performed by Miseq PE250, USA, and the platform of GenXPro (Frankfurt am Main, Germany) was used for sequencing. The analysis followed the QIIME2 pipeline along with Emperor program scripts. Demultiplexed sequences from each sample were quality-filtered, trimmed, de-noised, and merged. Then, the chimeric sequences were identified and removed. As a final step, the clean sequences were mapped against the SILVA database (v138) for bacterial taxonomic annotation. All raw sequences related to this experiment were deposited into the Short Read Archive (SRA) database of the NCBI under the Umbrella (multi-domains) BioProject accession number PRJNA986119.

### 3.7. Statistics

The bacterial taxa were classified using log-ratio-normalized counts. The relative abundance was mapped into a heat map based on ANOVA results where a *p*-value < 0.05 is considered significant. The individual taxa were represented using a color code of center-scaled mean read counts of three replicates. We plotted a Venn diagram to distinguish between core and specific bacterial features in different samples. We prepared Krona charts in interactive mode to present the bacterial relative abundance measured in bulk and rhizosphere soils.

## 4. Discussion

For better understanding of olive–microbe specificity, we tested the biochemical feedback in contrasting olive genotypes maintained under different conditions. Overall, the olive seedlings exhibited an intraspecific variability in the ability to withstand drought stress. Genetics played a major role in tailoring cultivar phenotypic expressions [27]. Many indicators have drawn the plant response to abiotic perturbation [28]. Among these indicators, the biochemical pathways are the most involved in plant homeostasis [29]. Thus, we applied metabolic profiling to decipher the mediation of endospheric microbes in the plant-specific response to drought. The analysis of metabolic features demonstrated contrasting results. Dissimilarities in leaf metabolic composition were genotype-dependent. Several classes of primary and secondary metabolites were identified in foliar extracts. Drought stress induced a high accumulation of carbohydrate compounds in both varieties, more specifically in the sensitive genotype. An increased production of sugar alcohols and water-soluble sugars (glucose and sucrose) was mainly detected in roots of the Chetoui cultivar inoculated with their conspecific microbiome. These results indicate that the accumulation of these osmolytes can be part of a microbe-adaptation mechanism rather than degradation products of a stressed cultivar [30]. This adaptation mechanism consists in a plant minimizing the allocation of carbohydrates to sink organs to increase their concentration in leaves, which would decrease the energy dissipated during the photosynthesis process [31]. In accordance with our results [32], it was found that a wheat drought-sensitive genotype (AGS2038) compromised with more resource allocation to leaves, instead of roots, when exposed to drought stress. The identified molecules are sucrose, galactinol, mannitol, diglycerol, glucose, galactose, inositol, and sorbitol and are essential constituents of vegetative tissue [33]. They serve as stress osmoreceptors, maintain cell turgor, and provide hydration around proteins [34], hence modulating the plant growth and physiology [31]. Furthermore, sugars and their derivatives are considered potential signaling molecules. They coordinate symbiotic interaction to support plant needs in water and nutrients [35]. For instance, study [36] showed evidence that carbohydrates can affect all phases of the life cycle of *Suaeda nudiflora* wild mosque plants and their symbiotic bacteria Bacillus megaterium and Pseudomonas aeruginosa. Besides carbohydrates, high concentrations of fatty acids were found in the seedlings of the Chemleli cultivar inoculated with wet-adapted consortia. Stearic, palmitic, and linoleic acids were slightly accumulated in the Chetoui variety in the stressed, non-inoculated treatment. These plastid-generated lipids act as a hydrophobic barrier for leaf cell walls. They protect the membrane from damage [37], regulate the phytohormone biosynthesis [38], and facilitate the transport of solutes under abiotic stress [39]. The plant genotype and water state are partially responsible for lipidomic profile remodeling in many crop tissues including olive organs [40]. Symbiotic bioagents colonizing plant roots were also proven to engender strike modification in fatty acid structure and composition [41], specifically in their related host. Lipids have diverse functions in regulating the first barrier of the plant-root–microbe interface represented by the plasma membrane. They serve as signaling molecules exchanged during the recruitment process [42]. With metabolic fingerprinting, we inspected several classes of secondary metabolites; for a wide range of phenols and methoxyphenols, the tolerant variety displayed altered patterns among the four treatments. Yet, the endophytic consortium colonizing the Chetoui variety stimulates a strong accumulation of 3,4-Dimethyphenol, 9,12,15-Octadecatrienoic acid, syringylacetone, benzoic acid, 2-Methoxy-4-vinylphenol, and 3,4-Dimethyl benzoic acid in inoculated treatments of the well-watered condition. In a previous study, [43], the soil PGPRs were found to increase plant total phenolic content in olive species [44]. The drought-sensitive genotypes form phenolic molecules to tackle tissue desiccation [45]. Hence, the biosynthesis and accumulation of phenols can be regarded as indicators of drought resistance in susceptible cultivars [46]. The obtained results suggest that the leaf metabolome of the tolerant variety subjected to external factors is deemed to be stable owing to its intrinsic robustness [47]; however, the foliar chemical composition of the sensitive variety was very susceptible to stress and biostimulant application. Leaf metabolic traits associated with plant intrinsic plasticity were found to modulate the long-term drought response in various crops [48]. Substances like stilbenoids and tocopherols were mainly accumulated in the stress condition. Under drought, one molecule of alpha tocopherol is thought to be able to deactivate 120 molecules of oxygen to protect the cellular membrane [49]. The RNAi-mediated disruption of a rice farnesyltransferase/squalene synthase (SQS) by maize squalene synthase also improved drought tolerance in vegetative and reproductive stages [50]. Venn diagram results (Figure 4) combined with the heat map results (Figure 1) revealed significant metabolic biomarkers that can be considered as a common core. Around 60% of the identified metabolites belonging to fatty acids (palmitic acid, linoleic acid, arachidic acid), sugar (sucrose, D-mannitol, diglycerol, 1,6-anhydro-glucose, 1,5-anhydro-D-sorbitol), and phenol class (caffeic acid, 4-hydroxycinnamic acid, 3,5-dimethoxy-4-hydroxycinnamic acid, phytol) with tocopherol were shared among the treatments of the tolerant variety, which preserved a relatively stable metabolic pattern during the experiment. Regardless of stress or inoculum application, compounds like oleanolic acid, epicatechin, ursolic acid, D-glucose or D (+) galactose, and galactinol were depleted. Molecules of 3,4-Dimethyphenol, 9,12,15-Octadecatrienoic acid, syringylacetone, benzoic acid, 2-Methoxy-4-vinylphenol, and 3,4-Dimethylbenzoic acid and coniferyl alcohol also demonstrated decreased concentrations. However, organic acids like succinic, linoleic, arachidic, and caffeic acids conserved a highly abundant profile in the four treatments of the Chemleli cultivar. Distinguished behavior can only be detected when the tolerant variety is maintained under a well-watered condition and inoculated with wet-adapted consortia where the leaves tend to strongly accumulate specific metabolites like 3,5-dimethoxy-4-hydroxycinnamic acid, Rhapontigenin, diglycerol, behenic acid, 4-vinylphenol 2, trans-13-octadecenoic acid, 1,6-anhydro-glucose. Drought stress and PGPR colonization appear to induce significant perturbation in phytochemistry of the sensitive cultivar. A small fraction of metabolites comprising trans-13-octadecenoic acid, stearic acid, palmitic acid, linoleic acid, and alpha tocopherol was shared between Chetoui treatments. Many studies illustrated the presence of a common core metabolome between genotypes of the same plant that might be preserved at the species level [51]. In this regard, it has been proven that the composition, the core-stress-responsive metabolome, and their integral role in the plant system are conserved regardless of the plant tolerance degree [52]. In the optimum condition, non-stressed seedlings seemed to be enriched in sugars and linoleic acid compounds in the absence of the inoculum. The introduction of PGPR consortia to the Chetoui endosphere strongly affected the leaf biochemistry of the sensitive cultivar by decreasing sugar and linoleic acid concentration and raising 3,4-Dimethyphenol, 9,12,15-Octadecatrienoic acid, syringylacetone, benzoic acid, 2-Methoxy-4-vinylphenol, and 3,4-Dimethylbenzoic acid and coniferyl alcohol to a maximum level. Under stress, the rhizospheric inoculum drove the plant to further accumulate alpha tocopherol, squalene, and quinic acid. Plant-associated microbes are endowed with high capacity to influence the plant response to abiotic stress through supporting different metabolic mechanisms like inducing osmo-regulator production [53]. Assessing microbe-mediated change in the olive leaf metabolome of seedlings subjected to drought stress would give insight into the genotype-specific response of crops facing water deficiency [54]. Although the role of beneficial microbes in regulating the metabolism of their plant partner is well documented [55], the effect of host–microbe preferences in modulating the outcomes of the symbiosis remains unclear [56]. In this regard, we attempted to compare the effect of specific microbial consortia on their local cultivar and a distant variety growing in a different environment. Amplicon sequencing data revealed a genotype-oriented segregation of bacterial taxa. The two cultivars followed different recruitment strategies in watered and non-watered conditions. It can be seen (Figure 2) that at the phylum level, drought can negatively affect the abundance of Proteobacteria (Alpha- and Betaproteobacteria) in the endosphere of the Chetoui cultivar. However, it is worth noting that Pseudomonas was the most dominant taxon to gather around the roots of both varieties regardless of normal watering or the stress condition. Drought brings positive effects for the firmicute phylum, which leads to an increase in the abundance of Bacilli and Clostridia treatments in the Chemleli variety, suggesting that drought has a more favorable environment for the proliferation of the Terrabacteria group and that PGPRs like *Bacillus, Rhizobium, Staphylococcus, Bifidobacteruim, Sphingomonadales, and Microcaccales* genera are endowed with better drought resistance, leading to the reduction in negative effects on plants [55]. At the species level, the uneven abundance of *Thermosipho africanus, Alkalihalobacillus alcalophilus, Paenibacillus alvei, Caldanaerobius polysaccharolyticus, Mycobacterium conspicuum, Streptomyces macrosporus, Prauserella rugosa, Pseudomonas alcaligenes, and Sphingobacterium comitans* between stressed and well-watered treatments can reflect the different trend in microbial community recruitment followed by two cultivars. In our study, *Proteobacteria* constitute a part of the olive core microbiome since this family was recruited by both genotypes in the root compartment; *Bacillaceae,* Pseudomonadaceae, and *Rhizobiacea* shared among treatments represent other families in the core prokaryotic community. *Pseudomonas* are ubiquitous in nature and include resilient species able to survive the extreme environments; they colonize the soil and root endosphere [57]. Members of this family are diverse; they are mainly represented by *Acinetobacter* species. On the other hand, several taxa of the *Bacillalecea, Fervidobacteriaceae,* and *Bacteroidaceae* family are used as biofertilizers [56]. The tolerant variety appeared to acquire more beneficial microbes from the bulk soil rather than the conspecific microbiome of another accession. Indeed, the highest affinity between symbiotic partners was frequently described in experiments when the local host and their indigenous microbiome shared the same habitat prior to soil sampling [57]. Genome-wide studies identified plant loci responsible for controlling the heritability in rhizosphere microbial communities [58]. For instance, the authors of [59] examined the role of the plant type and soil composition in coordinating the microbial community succession and stability over plant generation. It was proven that plants exerted a selection pressure during the assembly of complex microbial consortia based on their capacity to support the partner fitness [60]. Optimum water status seemed to induce high bacterial proliferation in the roots of the drought-sensitive variety placed in contact with the rhizosphere consortium or with bulk soil. Despite the variability in enriched and depleted bacterial profiles of well-watered treatments, microbes were firmly adapted to a specific water regime. These findings align with [61], which revealed that soil microbes could increase plant growth maximumly when pairing with a host that matches their historical moisture state. Information concerning the microbial moisture history of a holobiont is consistently explored to engineer resilient synthetic communities [62]. When the Chetoui cultivar experienced low water potential, the abundance of their root endophytes declined to critical levels. Some plant species adopted an avoidance strategy to restrict supplying their symbiotic partners with photosynthetic reserves. The previously accumulated products would be slowly mobilized during the stress period [63]. Susceptible genotypes perform a sanction-based strategy when microbial infection burdens their functioning [64]. Thus, microbial population tradeoff remains a condition of the original inoculum characteristics [65], mainly the previous moisture state [66]. Distinct bacterial features were found in the root samples of well-watered treatments. When seedlings were planted in soil obtained from an olive grove, the Chemleli variety strongly accumulated PGPRs from the bulk soil; however, the Chetoui endosphere was mostly colonized by conspecific bacteria from the rhizosphere. According to [67], the microbial community assembly follows two modalities: In the first modality, the microbes assemble through rhizosphere recruitment if the inoculum is related to the host (the example of drought-sensitive cultivar). In the second modality, the microbiome assembles through stochastic dispersal of the bulk soil reservoir when the inoculum and host are genetically distant (the example of olive-tolerant variety). Results of the physicochemical characterization of the microbial source used in our experiment yield significantly different results. In the rhizosphere, microbes operating in close interaction with the host presented higher nutrient and microbial abundance than the bulk soil. This is partially determined by the plant species identity. The shared microbial taxa found in the endosphere of both varieties can be explained with reference to the common bacterial taxa resident in bulk and rhizosphere soils obtained from the same site [68]. Some species found in the root compartment of olive seedlings are not acquired from the inoculum source. They might be recruited from other environments like water, air, etc. [69], thus defining the inoculum origin and proprieties that can help us understand the plant selection strategy of microbial inocula.

The application of the indigenous microbiome of the Chetoui variety changed their leaf metabolic profile to match the foliar biochemistry of the drought-tolerant seedlings maintained under the well-watered condition. Seemingly, within three generation experiments [61], it was found that the maximal microbiome efficiency in ameliorating plant growth and physiology can be reached only when the watering condition of the host plants resembles their corresponding microbes.

## 5. Conclusions

Microbial symbiosis is considered an adaptative process and source of phenotypic complexity in plants. Microbes regulate the biosynthesis of leaf metabolites to enhance plant resistance to drought. In this study, we analyzed the genotype-mediated change in root endophytes and the leaf metabolic profile of contrasting olive genotypes subjected to drought stress. The phytochemistry of the sensitive variety appears to be more susceptible to drought and microbial application. PGPR induced significant modifications in leaf metabolic patterns matching the phytochemistry of the drought-tolerant cultivar maintained under optimum conditions. The wet-adapted inocula seem to provide more benefits to their local host compared to a distant variety. The Chemleli cultivar was slightly affected by the genetically distant microbiome obtained from a contrasting environment. Therefore, initial community composition, host identity, and the origin and characteristics of the soil source are deemed to be major factors to underpin microbial functioning. Dissimilarities in foliar metabolome composition depended basically on plant susceptibility to drought and their relatedness to microbial consortia. Resistant and sensitive cultivars attempted to recruit different bacterial species when challenged by drought stress, which means that plants employ variety-specific strategies to select beneficial soil bacteria to mitigate stress. Together, these results suggest that the characterization of plant–soil–microbe interaction likely enable the genetic engineering of resilient crops.

## Figures and Tables

**Table 1:** Physicochemical characteristics of rhizosphere and bulk soils.

Sample	C (g/kg)	TN (mg/kg)	Available P (mg/kg)	EC (mS)	pH	H (%)
Rhizosphere	18.98 ± 1.65	85.21 ± 13.42	63.34 ± 5.12	150.16 ± 2.46	7.40 ± 0.5	87 ± 2.75
Bulk soil	5.05 ± 3.79	57.10 ± 4.06	54.52 ± 12.32	90.07 ± 6.25	8.16 ± 0.2	74 ± 3.32

## Data Availability

Data is contained within the article.
